# 肺类癌的外科治疗及预后分析

**DOI:** 10.3779/j.issn.1009-3419.2019.08.03

**Published:** 2019-08-20

**Authors:** 少伟 张, 志强 薛, 佳新 温, 波 王, 向阳 初

**Affiliations:** 100853 北京，中国人民解放军总医院胸外科一病区 Department of Thoracic Surgery, PLA General Hospital, Beijing 100853, China

**Keywords:** 肺肿瘤, 类癌, 手术, 预后, Lung neoplasms, Carcinoid tumor, Surgery, Prognosis

## Abstract

**背景与目的:**

肺类癌是一种少见的神经内分泌肿瘤，有关其预后因素的研究较少。本研究旨在探讨外科手术治疗肺类癌的疗效，并分析其预后影响因素。

**方法:**

回顾2000年1月-2017年12月解放军总医院收治的肺类癌患者的临床资料。以*Kaplan-Meier*方法计算患者的生存率，比较不同因素对预后的影响，进行单因素分析，并采用*Log-rank*检验; 通过*Cox*比例风险模型进行多因素分析。

**结果:**

98例肺类癌患者行外科手术治疗，其中典型类癌和不典型类癌者分别41例、57例。肺类癌患者的5年及10年总体生存率分别为80.0%、73.6%。单因素分析显示，年龄(*P*=0.000, 1)、吸烟(*P*=0.005)、病理亚型(*P* < 0.000, 1)、肿瘤分期(*P* < 0.000, 1)、T分期(*P*=0.000, 2)是影响预后的因素。多因素分析显示，年龄(*P*=0.005)、肿瘤分期(*P*=0.017)是预后的独立影响因素。

**结论:**

行外科手术治疗的肺类癌患者总体预后较好。年龄、肿瘤分期是影响肺类癌术后远期生存的独立危险因素。

## Abstract (en)

**Background and objective:**

Bronchopulmonary carcinoid (BPC) is a rare neuroendocrine tumor, the clinical studies on treatment and prognostic factors of BPC are somewhat controversial. Our purpose was to evaluate the clinical efficacy of surgery on BPC, and to analyze the prognostic factors affecting the survival of BPC.

**Methods:**

We retrospectively collected the clinical data of patients with BPC admitted to the Chinese PLA General Hospital between January 2000 and December 2017. The *Kaplan-Meier* method was used to calculate the survival rate of patients and to map the survival curve. Then the effects of different factors like pathological classification, gender, age, on prognosis were compared by univariate analysis and multivariate analysis was made by *Cox* proportional hazard model.

**Results:**

A total of 98 patients had a diagnosis of BPC were included in the study. There were 41 patients with typical carcinoid (TC) and 57 patients with atypical carcinoid (AC). The 1-yr, 5-yr, and 10-yr overall survival rates of BPC were 96.9%, 80.0% and 73.6%, respectively. Univariate analysis showed age (*P*=0.000, 1), smoking history (*P*=0.005), pathological subtype (*P* < 0.000, 1), T stage (*P*=0.000, 2), TNM stage (*P* < 0.000, 1) were the prognostic factors. Multivariate analysis showed that age (*P*=0.005) and tumor stage (*P*=0.017) were independent prognostic factors.

**Conclusion:**

BPC occurred more in middle-aged men. Surgery is the main treatment for lung cancer, and the overall prognosis is good. Age and TNM stage were independent risk factors for long-term survival after lung cancer surgery.

肺类癌起源于支气管黏膜下Kulchitsky细胞，是一种分化良好的神经内分泌肿瘤，占全身所有类癌的1/4-1/3，分为典型类癌(typical carcinoid, TC)和不典型类癌(atypical carcinoid, AC)^[1, 2]^。有研究^[1, 3]^表明肺类癌发病率呈持续上升趋势，外科手术是唯一能够治愈局限期肺类癌的手段。但是关于肺类癌的生存率及影响肺类癌术后远期生存因素的研究较少，且不同研究之间有一定争议^[3-6]^;本研究旨在探讨外科手术治疗肺类癌的疗效，并分析影响肺类癌手术后生存的主要因素。

## 1 材料与方法

### 1.1 临床资料

收集中国人民解放军总医院2000年1月-2017年12月期间收治的肺类癌患者的病历资料。首先，在电子病历系统中将上述日期内所有患者定为检索范围; 其次，分析电子病历系统中肺类癌适用的通用名，并进行检索; 再次，记录发病部位为支气管、肺以或不明确病历的病案号; 最后，复核上述病历，排除未进行外科手术、诊断不明确、发病部位不是肺或支气管、复合型神经内分泌肿瘤、数据缺失以及拒绝随访或未曾进行过随访的患者。收集患者年龄、性别、手术方式、肿瘤分期等信息。

### 1.2 病理诊断

由两名经验丰富的病理科专家分别阅片审核并给出诊断。以世界卫生组织(World Health Organization, WHO)肺肿瘤分类为标准^[7]^，将每2 mm^2^核分裂象小于2个，肿瘤直径 > 5 mm，且无坏死的类癌诊断为TC; 将每2 mm^2^核分裂象介于2个-10个，无坏死或伴有点状坏死的类癌诊断为AC。根据国际肺癌研究协会(The International Association for the Study of Lung Cancer, IASLC)第8版肺癌原发灶-淋巴结-转移(tumor-node-metastasis, TNM)分期标准对肿瘤进行分期。

### 1.3 随访

通过电话、调查问卷等方法对患者进行随访，获取其生存信息。所有患者均随访到死亡或2019年1月31日为止。生存期(overall survival, OS)以月为单位计算。截止到随访日期结束，未出现观察终点的病例及失访病例均按删失数据处理。

### 1.4 统计学方法

采用统计软件R-3.4.0进行数据处理分析。描述性统计中，定量变量用Mean±SD或中位数表示，用卡方检验或*Fisher*精确检验，定性变量用频数和百分比表示; 使用*Kaplan-Meier*方法计算生存率并绘制生存曲线，单因素分析采用*Log-rank*检验; 使用*Cox*比例风险模型进行多因素分析。本研究中，所有检验均为双侧检验，检验水准*P* < 0.05。

## 2 结果

### 2.1 一般资料

最终纳入98例肺类癌患者行外科手术治疗，其中TC、AC患者分别为41例、57例。总体男女比例为1.88:1，中位年龄53.5岁，平均年龄(53.27±13.00)岁; TC较AC平均年龄低，AC更多见于男性，吸烟患者比例更高35例患者无临床症状通过体检发现病变; 63例有临床症状，其中有13例患者至少出现1次阻塞性肺炎，常见临床症状有咳嗽咳痰(41.84%, 41/98)、痰中带血或咯血(26.53%, 26/98)、胸闷气促(17.35%, 17/98)、发热(9.18%, 9/98)。1例合并异位促肾上腺皮质激素分泌(adrenoeorticotropic hormone, ACTH)综合征，出现胡须增多、色素沉着、下肢水肿等Cushing综合征表现。

### 2.2 肿瘤特征

肿物位于右肺上叶、中叶、下叶、中间干支气管分别11例(11.22%)、8例(8.16%)、22例(22.45%)、7例(7.14%)，左肺上叶、下叶分别18例(18.37%)、29例(29.59%)，左、右主支气管分别2例(2.04%)、1例(1.02%)。肿瘤最长径范围0.6 cm-7.3 cm，平均3.0 cm，其中3例患者为双病灶。

### 2.3 手术情况

所有纳入患者均行外科手术治疗，其中肺叶切除术60例(61.22%)、联合肺叶切除12例(12.24%)、全肺切除术11例(11.22%)、支气管袖式切除术3例(3.06%)、肺段2例(2.04%)、楔形切除10例(10.20%)。90例(91.84%)行淋巴结清扫或采样手术。本组无术中死亡病例，术后2个月内死亡2例，均为患不典型肺类癌的高龄男性(73岁、76岁)，术式分别为左上肺楔形切除术、左上肺叶切除术。21例(21.43%)患者术后或术前曾行化疗。

### 2.4 肿瘤分期

Ⅰ期65例(66.32%)，Ⅱ期26例(26.53%)，Ⅲ期7例(7.14%)。淋巴结转移共14例(14.29%)，其中N1期7例，N2期7例。脉管癌栓9例，切缘阳性3例。AC相较于TC，更容易出现淋巴结转移(11/57 *vs* 3/41，*P*=0.143，*Fisher*精确检验)，更倾向于出现脉管癌栓(8/57 *vs* 1/41，*P*=0.075，*Fisher*精确检验)。

### 2.5 随访及生存情况

中位随访时间为54(1-216)个月，中位生存期未达到。本组98例患者中，死亡19例，有2例于术后2个月内死亡，除上述2例外，死亡患者中位生存期24(12-98)个月。98例肺类癌手术患者的1年、5年及10年总体生存率分别为96.9%、80.0%及73.6%(图 1)。其中AC患者57例，1年、5年及10年总体生存率分别为94.6%、65.3%及60.6%。TC 41例，死亡1例，发生在术后98个月，其1年、5年及10年总体生存率分别为100.0%、100.0%及90.9%(图 2)。

**1:**
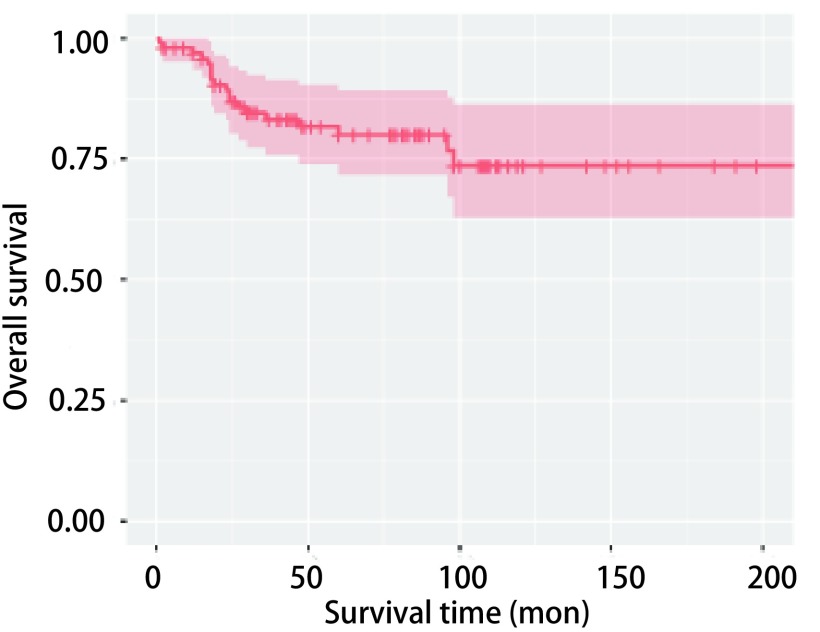
98例肺类癌患者的总体生存曲线 *Kaplan-Meier* curves for 98 patients with bronchopulmonary carcinoid

**2:**
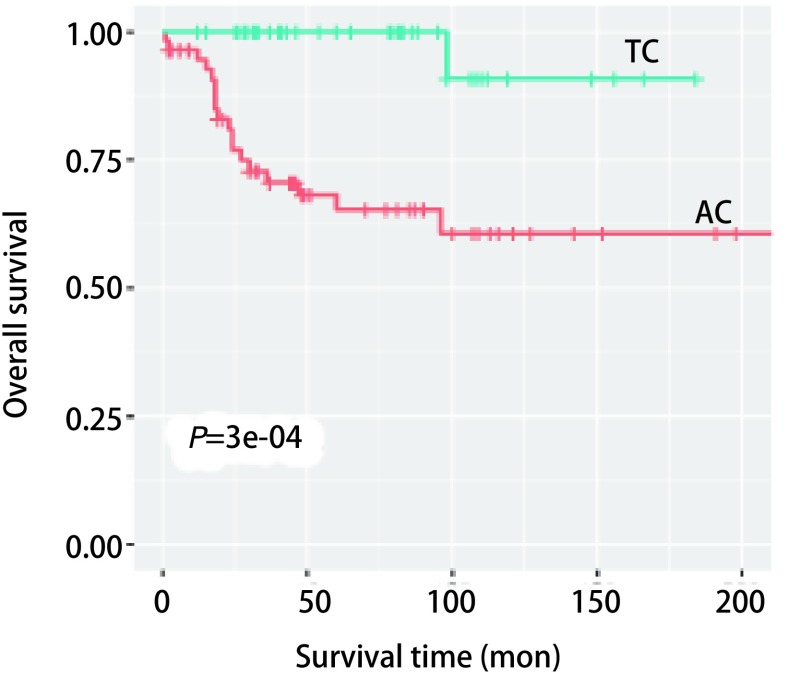
典型类癌与不典型类癌患者生存曲线比较 *Kaplan-Meier* curves for bronchopulmonary carcinoid according to pathological subtype

### 2.6 预后因素分析

分别以性别、年龄、是否吸烟、病理亚型、手术方式、淋巴结处理方式、淋巴结是否转移、肿瘤分期及化疗等因素进行分组，比较各组患者的生存差异，进行单因素统计分析，并进行*Log-rank*检验，结果提示病理亚型、年龄、吸烟、肿瘤分期、T分期与预后相关，生存差异有统计学意义。详见表 2。将单因素分析有统计学意义(*P* < 0.05)的变量：年龄、吸烟史、病理亚型、肿瘤分期(T分期由于与肿瘤分期存在共线性，故未纳入多因素模型)，纳入*Cox*回归模型进行多因素分析，结果显示：年龄、肿瘤分期是影响预后的独立因素，年龄大、肿瘤分期晚的患者，预后较差，详见表 3。

**1:** 98例肺类癌患者的一般资料 Demographics of 98 patients with bronchopulmonary carcinoid

Characteristics	TC	AC	Total cohort	*P*
*n*	41	57	98	
Age (yr)				
Mean±SD	46.63±11.85	58.04±11.62	53.27±13.00	< 0.001
Range	18-74	27-77	18-77	
Gender				0.005
Male to female	20 (48.8%): 21 (51.2%)	44 (77.2%): 13 (22.8%)	64 (63.5%): 34 (34.7%)	
Smoking index				0.014
> 400	6	23	29	
≤400	8	11	19	
0	27	23	50	
Asymptomatic	15	20	35	1.000

**2:** 肺类癌患者预后的单因素分析 Univariate analysis of prognosis for 98 patients with bronchopulmonary carcinoid

Index	*n*	Death toll [*n*(%)]	Survival probability		*P*
			5 years	10 years	
Pathological type					< 0.000, 1
TC	41	1 (2.44)	1.000	0.909	
AC	57	18 (31.58)	0.653	0.606	
Gender					0.237
Male	64	15 (23.44)	0.773	0.682	
Female	34	4 (11.76)	0.853	0.853	
Age (yr)					0.000, 1
< 55	52	3 (5.77)	0.958	0.898	
≥55	46	16 (34.78)	0.609	0.542	
Smoking history					0.005
Smoking	48	15 (31.25)	0.709	0.600	
No-smoking	50	4 (8.00)	0.890	0.890	
Symptom					0.175
Asymptomatic	35	4 (11.43)	0.878	0.878	
Symptomatic	63	15 (23.81)	0.764	0.668	
Hypertention					0.335
Yes	23	6 (26.09)	0.819	0.585	
No	75	13 (17.33)	0.793	0.793	
TNM stage					< 0.000, 1
Ⅰ	65	5 (7.69)	0.906	0.906	
Ⅱ/Ⅲ	33	14 (42.42)	0.588	0.441	
T stage					0.000, 2
T1	53	3 (5.66)	0.960	0.896	
T2	32	10 (31.25)	0.672	0.588	
T3/4	13	6 (46.15)	0.480	0.480	
N stage					0.118
N0	84	14 (16.67)	0.824	0.785	
N1/2	14	5 (35.71)	0.638	0.479	
Surgery					0.499
Lobectomy	63	12 (19.05)	0.826	0.734	
Pneumonectomy	22	3 (13.64)	0.831	0.831	
Sublobar resection	13	4 (30.77)	0.645	0.645	
Chemotheraphy					0.109
Yes	21	7 (33.33)	0.699	0.524	
No	77	12 (15.58)	0.831	0.831	

**3:** 肺类癌患者预后多因素*Cox*风险比例模型分析 Multivariate *Cox* proportional hazards model analysis of prognosis for lung carcinoid

Variates	Coefficient (*β*)	SE	*P*	HR (95%CI)
Age (every additional year)	0.072	0.026	0.005	1.075 (1.022-1.131)
Smoking history (Non-smoking)	0.549	0.587	0.349	1.732 (0.548-5.468)
Pathological type (TC)	-1.791	1.034	0.083	0.167 (0.022-1.264)
TNM stage (Ⅱ/Ⅲ)	1.263	0.528	0.017	3.537 (1.258- 9.949)

## 3 讨论

类癌是一种少见的、生长缓慢的神经内分泌肿瘤，可发生于胃肠道、肺、胰腺、胸腺等多种器官^[8]^。肺类癌发病率由1973年的0.3/10万上升至2004年的1.35/10万，其占全身类癌的构成比也在上升并稳定在25%-30%，呼吸系统是最常发生类癌的部位之一^[1, 2, 8]^。多数研究认为，类癌占肺原发恶性肿瘤的构成比为1%-2%^[3, 8]^。

目前认为，外科手术是唯一能够治愈局限期肺类癌的治疗手段。标准术式同非小细胞肺癌(non-small cell lung cancer, NSCLC)一样，在满足完全切除的前提下，采取解剖性肺叶/肺段切除加淋巴结清扫术，局限于主支气管的肿瘤优先考虑袖式切除手术，肺功能受限的患者，可采取姑息性手术^[3, 9]^。N2淋巴结转移不是手术的绝对禁忌证^[10]^。近年来，有许多学者在探索肺类癌的最佳切除范围，多关注肺叶切除与亚肺叶切除的生存差异，另有学者^[11-13]^研究了切缘与预后的关系，认为距切缘不足1 cm的AC预后较差，且亚肺叶切除是AC局部复发的独立危险因素。本研究比较了肺叶切除(含袖式切除)、亚肺叶切除、联合肺叶(含全肺)切除等不同切除范围患者的生存数据，结果显示不同亚组之间OS无统计学差异。这与Furqan等^[14]^的结果相一致，提示我们，在术前能确诊肺类癌的前提下，由于术式对远期预后无明显差异，而亚肺叶切除或袖式切除可保留更多肺功能，值得考虑更多地应用于肺类癌患者。但是，包括支气管镜活检在内的术前活检与术中冰冻均难以明确诊断肺类癌^[15, 16]^。也就是说，保留更多肺功能的手术方式更有针对性地应用于肺类癌患者还存在一定困难。

本研究中，AC相较于TC，更容易出现淋巴结转移(*P*=0.143)，也更倾向于出现脉管癌栓(*P*=0.075)，但均无统计学差异。其中，AC更容易出现淋巴结转移可能与AC侵袭性较强有关，文献[17]已有报道; 而脉管癌栓情况由于回顾性研究及病例数的限制，需要进一步研究去证实。值得注意的是，本组围手术期死亡2例，术中、术后并未出现大量出血，而有血流动力学不稳定的表现，这可能与肿瘤短期内释放大量血清素、组胺、缓激肽等生物活性物质而引起血流动力学异常有一定关系。对于大型手术，欧洲神经内分泌肿瘤协会(European Neuroendocrine Tumor Society, ENETS)建议围手术期使用奥曲肽预防类癌危象的发生^[1]^。但是，也有学者认为预防性应用生长抑素类药物不能阻止类癌危象的发生，并主张需要进一步的研究来阐明生长抑素类药物预防类癌危象发生的机制^[18, 19]^。Cordon等^[20]^进行的一项前瞻性研究表明，尽管应用了奥曲肽，仍有35%的患者术中会出现类癌危象，提示我们：一方面，生长抑素类药物可能并不能阻止类癌危象的发生，另一方面，需要警惕术中发生类癌危象。

肺类癌对传统化疗药物并不敏感，有研究^[10]^总结了几个肺类癌化疗的研究，虽然不同研究的结果有一定差异，但样本量较大的两项研究均提示：相比于定期随访，化疗并没有提高TC/AC患者的无进展生存期和OS。一般来说，化疗可使肺类癌患者获得不高于30%的总体缓解率，目前多推荐化疗用于其他治疗失败的进展期AC^[1, 7]^。本研究中，化疗与未化疗者在总体生存期上并无显著差异，结果与已有报道一致^[14, 21, 22]^。此外，值得关注的是，2016年2月美国食品药品监督管理局(Food and Drug Administration, FDA)批准依维莫司用于失去手术机会晚期肺类癌的治疗^[3]^。

肺类癌的预后明显好于其他病理类型的肺癌，TC的预后又好于AC。不同研究的生存数据差异较大，TC和AC的术后5年生存率分别达90%-97%、44%-89%，10年生存率分别达56%-89.9%、47%-62%^[3-6]^;影响肺类癌预后的因素很多，包括病理分型、淋巴结转移、Ki-67指数、年龄、SUVmax值等，多数研究认为病理亚型及TNM分期是预后的影响因素^[4, 5, 23-25]^。另外，诸如淋巴结受累等因素是否是影响预后的独立危险因素，不同研究结论之间并不一致^[5, 17, 26]^，这可能与样本量大小、淋巴结清扫数目、病理亚型构成等有关。本研究中，单因素生存分析提示年龄、吸烟史、病理亚型、肿瘤分期、T分期为有统计学差异的预后影响因素，经*Cox*模型多因素分析提示年龄、肿瘤分期是影响预后的独立因素。Ki-67指数由于缺失值较多，且与病理亚型一致度较高，考虑存在多重共线性，未纳入预后因素分析，关于其与预后的关系，不同研究的结论并不一致，有学者根据病理分化程度和Ki-67指数将肺神经内分泌肿瘤分为高分化且Ki-67指数不大于20%组(A)、高分化而Ki-67指数大于20%组(B)、低分化组(C)，结果显示B组的2年及5年无病生存率低于A组且有统计学意义，而与C组表现无统计学差异^[23, 27, 28]^，笔者认为，确定其与预后关系可能需要更大病例样本的研究。

评价肺类癌的预后以及治疗的获益情况，笔者认为还需要关注疾病的自然病程。治疗的目的在于延长生存、改善生活质量，临床工作中，我们不仅要告知患者接受具体治疗措施后的获益情况，也要告知患者不接受此治疗的自然病程，但目前类似研究较少。Raz等^[13]^基于监测-流行病学及预后(Surveillance, Epidemiology, and End Results, SEER)数据库进行了一项回顾性研究，比较了TC手术治疗与非手术治疗的生存情况，纳入的4, 111例患者中，结果显示亚肺叶切除、肺叶切除的5年疾病特异性生存率(disease-specific survival, DSS)分别为98%、97%，另有306例患者未进行任何治疗，其5年DSS仍达88%，提示对于有高手术风险的患者，非手术治疗不失为一种选择。

一般认为肺类癌术后每年至少复查一次。如果未进行R0切除、分期较晚或以治疗为目的，随访间隔及检查项目宜进行个体化评估^[1]^。由于淋巴结阴性的TC患者预后很好且复发率极低，有学者主张术后随访可仅针对性地对淋巴结阳性和AC患者常规进行^[29]^。Marciello等^[30]^对AC患者进行了分析后认为，对于R0切除的AC患者，肿瘤复发多发生于术后5年内，淋巴结受累是复发转移的高危因素，需重点关注支气管、纵隔淋巴结、肝脏及骨骼可能发生的转移。我们通过随访发现，本组患者术后死亡均发生在100个月内，尤其是AC患者，提示术后8年内定期复查有助于及时发现肿瘤的复发转移。

总之，外科手术是治疗肺类癌的重要方法，行外科手术治疗的肺类癌患者总体预后较好。年龄、肿瘤分期是影响肺类癌术后远期生存的独立危险因素。
